# Halogenated By-Products in Chlorinated Indoor Swimming Pools: A Long-Term Monitoring and Empirical Modeling Study

**DOI:** 10.1021/acsomega.3c00091

**Published:** 2023-03-15

**Authors:** Mesut Genisoglu, Mert Minaz, Ertac Tanacan, Sait Cemil Sofuoglu, Sehnaz Sule Kaplan-Bekaroglu, Amer Kanan, Nuray Ates, Tugba Sardohan-Koseoglu, Nevzat Özgü Yigit, Bilgehan Ilker Harman

**Affiliations:** † Department of Environmental Engineering, Izmir Institute of Technology, Izmir 35430, Turkey; ‡ Department of Environmental Engineering, Suleyman Demirel University, Isparta 32260, Turkey; § Department of Aquaculture, Recep Tayyip Erdoǧan University, Rize 53100, Turkey; ∥ Department of Environment and Earth Sciences, Al-Quds University, Jerusalem 51000, Palestine; ⊥ Department of Environmental Engineering, Erciyes University, Kayseri 38280, Turkey; # Department of Biomedical Engineering, Applied Sciences University of Isparta, Isparta 32200, Turkey

## Abstract

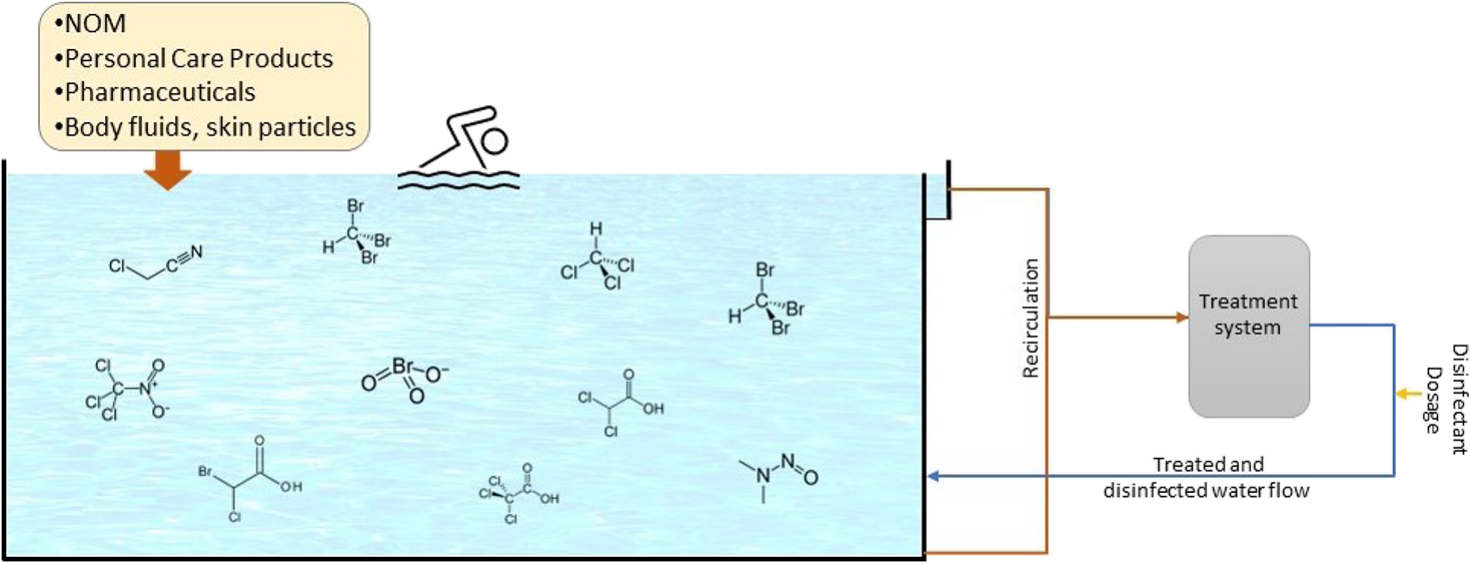

Monitoring the disinfection process and swimming pool water quality is essential for the prevention of microbial infections and associated diseases. However, carcinogenic and chronic-toxic disinfection by-products (DBPs) are formed with reactions between disinfectants and organic/inorganic matters. DBP precursors in swimming pools originate from anthropogenic sources (body secretions, personal care products, pharmaceuticals, etc.) or chemicals used in pools. Temporal (48 weeks) water quality trends of trihalomethanes (THMs), haloacetic acids (HAAs), haloacetonitriles (HANs), and halonitromethanes (HNMs) in two swimming pools (SP-A and SP-B) and precursor–DBP relationships were investigated in this study. Weekly samples were taken from swimming pools, and several physical/chemical water quality parameters, absorbable organic halides (AOX), and DBPs were determined. THMs and HAAs were the most detected DBP groups in pool water. While chloroform was determined to be the dominant THM compound, dichloroacetic acid and trichloroacetic acid were the dominant HAA compounds. The average AOX concentrations were measured to be 304 and 746 μg/L as Cl^–^ in SP-A and SP-B, respectively. Although the amount of AOX from unknown chlorinated by-products in SP-A did not vary temporally, a significant increase in unknown DBP concentrations in SP-B was observed over time. AOX concentrations of chlorinated pool waters were determined to be an important parameter that can be used to estimate DBP concentrations.

## 1 Introduction

Disinfection of water is a process applied since 1902 to neutralize pathogenic microorganisms in drinking water and prevent infectious diseases.^1^ While disinfectants/disinfection methods such as chloramine, ozone, chlorine dioxide, and ultraviolet radiation (UV) are used effectively, chlorine is the most preferred agent for disinfection of water.^2^ Chlorine-based disinfectants are generally used to reduce biological hazards caused by pathogenic microorganisms to people in the pool environment.^3^ Outbreaks associated with swimming pools are caused by viruses, bacteria, protozoa, and fungi in the pool that cannot be removed or disinfected. Waterborne viral diseases are often attributed to adenovirus, hepatitis A, norovirus, and echovirus. Chlorination is a cost-effective disinfection technique to ensure water safety and is effective against many pathogens, especially bacteria and viruses.^4^ Also, with the COVID-19 pandemic, viral disinfection has become important to prevent virus transfer between swimmers in pools. Commonly used disinfectants such as chlorine, chloramine, chlorine dioxide, and ozone react with organic components in the pools, generating disinfection by-products (DBPs).^5,6^

In addition to organic pollutants in filling water, continuous organic load from swimmers may increase the carcinogenic DBP formation potential in swimming pools due to the abundance of DBP precursors and continuous disinfection dosing. Therefore, DBP formation in swimming pools is a more remarkable issue than other disinfected waters such as drinking water.^3^ The first DBP group observed in swimming pools was reported to be trihalomethanes (THMs).^1^ Subsequently, Martınez et al.^7^ reported the presence of haloacetic acids (HAAs). Many other DBP classes such as haloaldehydes, haloacetonitriles (HANs), haloketones (HKs), halonitromethanes (HNMs), haloamides, and several aromatic halogenated DBPs have also been identified,^8,9^ reaching approximately 700 species.^10^ Analysis of samples collected from 54 swimming pools showed that the DBPs with the highest concentrations were THMs.^11^ However, there is a study in which the HAA concentrations were higher than those of THMs.^12^ Although many studies focused on chlorinated pools, a few studies also investigated DBPs, which are formed by bromine as a disinfectant. Also, over 100 DBPs were determined in several pools and spas that were disinfected by chlorine or bromine.^13^

The formation potential of DBPs depends on many environmental, operational, and swimmer-related factors,^14^ the first of which is the natural organic matter (NOM) content of filling waters. NOM in source waters is also one of the DBP precursors. Seawater is a specific source water, associated with increased DBP formation due to its considerable inorganic salt content.^15^ Operational factors such as reaction time, pH, and temperature affect the speciation and formation levels of DBPs.^16^ Finally, body fluids (BFs) released from human bodies such as urine, sweat, saliva, and personal care products used by swimmers, e.g., sunscreens, hair products, lotions/soaps, and cosmetics, give rise to the formation of DBPs in pool waters.^17,18^

People are mainly exposed to DBPs in drinking water via the ingestion route, whereas inhalation and dermal absorption routes may also be important in swimming pools. Inhalation is the dominant route for swimmers’ exposure to DBPs that have potential adverse health effects such as respiratory irritation and asthma.^19^ Chloramines, for example, have been reported to cause chronic toxic effects such as loss of voice, sore throat, sputum, and asthma in swimmers.^20^ Furthermore, some other DBPs, i.e., HAN, HKs, and *N*-nitrosamines, which are more cytotoxic and genotoxic than THMs and HAAs, have become emerging concerns.^21,22^ Studies have shown that pool water is more likely to be cytotoxic, mutagenic, and genotoxic than tap water.^21^

There is also a lack of studies that estimate DBPs in swimming pools without complex analytical procedures. Although many predictive models have been developed to estimate DBP concentrations in drinking waters, those in swimming pools are limited.^23−27^ Peng et al.^26^ took into account water quality parameters, did not consider adsorbable organic halide (AOX) and specific ultraviolet absorbance (SUVA) as independent variables, and did not involve the number of swimmers. Despite the importance of the subject, long-term monitoring studies are sparse in the literature. To our knowledge, this is the first long-term study in Turkey. This study investigated physical and chemical operational factors and filling water characteristics, as well as AOX levels in waters of two swimming pools for 48 weeks. Among the most common DBP groups in pools, THMs and HAAs, and the concentrations of nitrogenous DBPs (HANs and HNMs), which have high toxicity in even their low concentrations, were also monitored. In addition to monitoring parameters, this study aimed to study the levels of unknown carcinogenic halogenated DBPs that are likely to occur, based on AOX and targeted DBP analyses. This study will not only provide guidance to pool water engineers about the occurrence of chlorination by-products and those precursors but also allow them to estimate the concentrations of by-products that may occur with the help of established simple linear regression models without the need for complex chromatographic sample preparation, analysis, and expensive analytical instruments. This year-long monitoring/modeling study might be of help to public health mitigation efforts.

## 2 Materials and Methods

### 2.1 Sampling and Analytical Methods

Two swimming pools with different types of filling waters were monitored for a year. Filling water of SP-A was chlorinated water, while SP-B was filled directly from groundwater. The pools were completely re-filled before the monitoring campaign. The water samples were taken weekly at a depth of 20 cm from the water surface and 50 cm away from the overflow collectors. Samples were taken on Sundays for the sake of being conservative, which was determined to be the most crowded day based on the analysis of user data before the monitoring campaign.

pH, free and total chlorine, nitrate, nitrite, ammonia, and total nitrogen parameters were analyzed in the samples. Free and total chlorine and pH were measured during sampling. Nitrogenous compounds and total organic carbon (TOC) were analyzed within 1 day in the laboratory. In situ measurements were made using a WTW Multi 340i/Set instrument except for pH that was measured according to standard methods using a Mettler Toledo AG SG2 pH meter and free and total chlorine analyses that were performed using the Hach Pocket Colorimeter II (SM 4500G). Nitrite and nitrate analyses were performed in Suleyman Demirel University-Geothermal Research and Application Center Laboratory using ion chromatography (Dionex ICS-3000). Ammonia was analyzed using a Hach Lange DR5000 spectrophotometer with the 380 N Nessler method. Dissolved organic nitrogen (DON) was calculated by subtracting the total inorganic nitrogen from the total nitrogen values. UV analysis was performed with Shimadzu UV-1700 at a 254 nm wavelength according to SM 5910. TOC was analyzed using a TOC-L CPH Shimadzu TOC analyzer according to SM 5310B. Analysis of AOX was performed using an Analytik Jena multi X 2500 instrument according to the USEPA 1650 method. THM, HAN, HNM, and HAA analyses were performed after liquid–liquid extraction with Agilent 6890 GC-ECD. The measurement methods for DBPs were determined according to USEPA 552.3 for HAA and USEPA 551.1 for THM, HAN, and HNM. Information on the extraction and GC analysis methods is provided in the Supporting Information (SI 1).

### 2.2 QA/QC

Pool water samples were taken in pre-cleaned glass bottles. The samples were immediately moved to the laboratory in cooling bags after sampling. All glassware was cleaned with solvent prior to use. Extracted samples were analyzed as soon as possible. Also, GC-ECD-analyzed results were confirmed by analyzing random samples using GC–MS at the Environment and Cleaner Production Institute in Marmara Research Center of TUBITAK. Calibrations were performed daily for TOC, TN, and AOX analyses.

### 2.3 Statistical Analysis

Measurement results are presented as the mean values along with the standard deviations. The median, 25^th^, and 75^th^ percentile values are also presented in the plotted presentations. Kolmogorov–Smirnov test was used to assess normality of each variable (Table S1). Both Pearson and Spearman correlation coefficients were used because some of the variables fitted to the normal distribution (chloroform in SP-A and chloroform and DCAA in SP-B) while some did not fit (DCAA and TCAA in SP-A and TCAA in SP-B). Simple linear regression (SLR) models were developed to assess usefulness for prediction of THM, HAA, and HAN concentrations using SUVA, DON, TOC, and AOX as predictor variables.

## 3 Results and Discussion

### 3.1 Physico-Chemical Characterization

**Figure 1:**
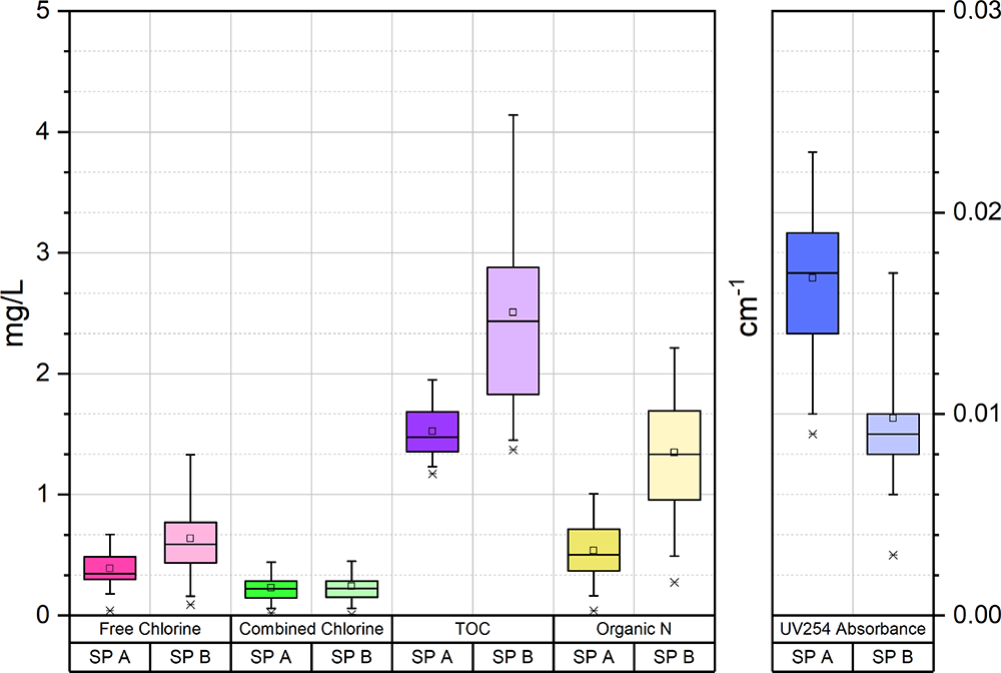
Physico-chemical characteristics of swimming pool waters.

Free and total chlorine, pH, UV_254_ absorbance, TOC, and DON parameters were monitored in swimming pool waters (Figure 1). The average pH values were determined to be 7.68 ± 0.19 and 7.49 ± 0.20 in SP-A and SP-B, respectively. At pH 7.5, the molar concentrations of HOCl and OCl ions were distributed equally. When the pH increases, the concentrations of the OCl ion increase while the concentrations of HOCl ions decrease.^28^ The optimum operating pH value is important in keeping balance between disinfection efficiency, piping and other equipment lifetime, and human health in swimming pools.^29^ At low pH values, efficient disinfectant HOCl is dominant. On the other hand, weak disinfectant OCl is the dominant species at high pH values, and the disinfection efficiency would be decreased. The determined pH values of both pools were ideal in terms of disinfection efficiency and human health. According to a review by Yang et al.,^30^ for many countries, these values do not fall out of acceptable limits. Although the electrical conductivities (ECs) measured for SP-A and SP-B were initially close, they increased more in SP-B than in SP-A with time (Figure S1). The increase in EC may be due to the chemicals used in the pool. In particular, those used for pH adjustment might be the cause of increasing EC. In addition, pool water was replaced with freshwater depending on the frequency of attendance. Hence, variables such as amount, concentration, and content of the pool chemicals may also be factors that influence the EC.

The average concentrations of free chlorine were determined to be 0.39 ± 0.16 and 0.64 ± 0.33 mg/L for SP-A and SP-B, respectively. The acceptable free chlorine concentrations in swimming pool water range from 0.3 to 0.6 mg/L in Germany,^31^ from 0.4 to 1.5 mg/L in Spain, from 0.4 to 1.4 mg/L in France, <3 mg/L in Switzerland, and from 0.3 to 0.5 mg/L in China.^32^ In Turkey, free chlorine concentrations in chlorinated indoor pool water are regulated to range from 1 to 1.5 mg/L.^33^ The amount of free chlorine decreases due to the consumption and decay of free chlorine and replacement of pool water during the intensive attendance in pools. Therefore, the free chlorine concentrations in the pool waters were determined to be variable. Due to continuous chlorination and organic material input, the combined chlorine for both pools increased depending on the time (Figure S2). The average combined chlorine concentrations were determined to be 0.23 and 0.24 mg/L in SPA and SPB, respectively. This increase of combined chlorine specifically indicates the formation of a number of disinfection products such as chloramine.

UV_254_ absorbance provides information about the type of NOM in water to indicate the presence of carbon–carbon bonds. A high UV_254_ absorbance means that NOM is hydrophobic and aromatic. It is also an indication for the high DBP formation potential in chlorination.^34,35^ Since a report by Edzwald et al.^36^ showing that there was a good correlation between THM formation and UV_254_, UV absorbance measurements in pools have been made and considered important because they give an idea about the aromaticity and hydrophilicity of organic substances in the water. The average UV_254_ values were determined to be 0.016 and 0.010 cm^–1^ in SP-A and SP-B, respectively. Compared to a study by Kanan,^37^ the UV_254_ absorbance for SP-A and SP-B is lower than in the pools of USA.

TOC concentration is important in revealing the DBP formation potential of disinfected waters.^38^ The average TOC concentrations were determined to be 1.52 ± 0.22 and 2.51 ± 0.87 mg/L for SP-A and SP-B, respectively. The highest TOC concentration was observed in August samples (week 43) probably due to high attendance (Figure S3). Spearman correlation analysis between the number of swimmers and TOC concentrations showed a positive relationship in both SP-A and SP-B (p < 0.01, Table S2). Although swimmer load in SP-A was higher than that in SP-B, TOC concentrations in SP-B were found to be higher than those in SP-A. This TOC profile can be due to the fact that swimmers in SP-A are more likely to be complying with hygiene rules, such as taking a shower before swimming or the efficiency of the treatment system in the water cycle. Peng et al.^39^ reported no correlation between TOC and the number of people attending the pool. Causes of increase in TOC concentration in SP-B can be due to (i) excessive use of pool chemicals, (ii) swimmer profile (age, gender, education level, etc.), (iii) increased amount of organic matter from the filling of pool water, (iv) not taking shower before pool attendance, and (v) low efficiency of the water treatment system. When two pools were compared in terms of swimmer profiles, approximately 92% of people attending SP-B were under 17 years. In particular, the higher TOC levels in SP-B in August can be due to the fact that the majority of the people attending the pool were younger-age groups (summer schools for pupils). In SP-A, it was observed that the majority of swimmers were academicians and university students (>18 years). The swimmers in SP-B being younger may probably lead to an increased amount of BFs into the water. Thus, participation of children to pools may lead to an increase in TOC and DBPs.^39^ Even the maximum TOC concentration in SP-A (2.05 mg/L) was determined to be lower than those reported in the literature with a range of 3.0–23.6 mg/L.^37,39−41^

DON concentration is essential for the formation of nitrogenous DBPs.^42^ The average DON concentration of SP-A was determined to be 0.54 ± 0.27 mg/L. In spite of a higher pool attendance, DON concentrations of SP-A were determined to be lower than those in SP-B (1.35 ± 0.54 mg/L), which might be due to the differences in the water replacement strategies, efficiency of the treatment system, and/or the swimmer profile. In addition, DON concentrations of the filling water of SP-A were determined to be lower than those in SP-B. From the beginning of the monitoring campaign, DON concentrations of SP-B were determined to be increased higher than those in SP-A (Figure S4). This phenomenon shows that the swimmer profile and/or the efficiency of the treatment system might significantly affect the DON concentrations in SPs. During the monitoring campaign, an increase in DON concentration was observed after the 41^st^ week. The average DON concentrations were 0.47 and 0.66 mg/L in the first 4 weeks, whereas they were 0.71 and 2.04 mg/L in the last 4 weeks in SP-A and SP-B, respectively. This is probably because swimmer load in the pools during the summer vacation period was two- to threefold higher than in the other periods.

### 3.2 AOX and DBP Concentrations

**Figure 2:**
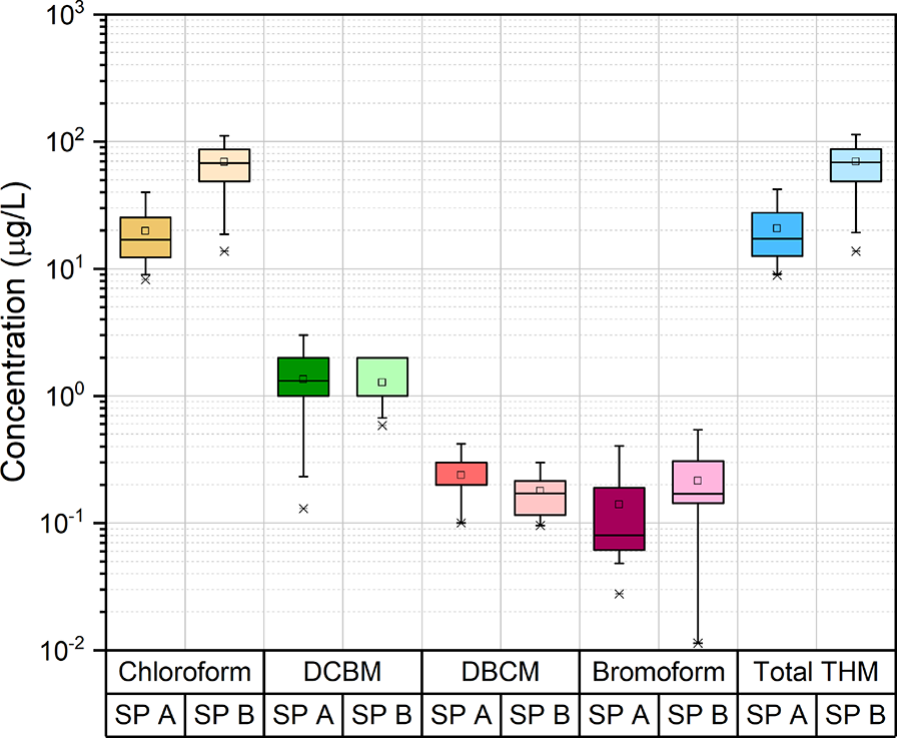
THM concentrations of swimming pool waters.

A total of 19 DBP species (THM, HAA, and HAN species) and AOX were monitored in the pools. While the detection frequencies of THMs and HAAs were determined to be 100%, HNMs were not detected. Also, the detection frequencies of HANs in SP-A and SP-B were determined to be 77.1 and 95.8%, respectively. The average THM concentrations of SP-A and SP-B were determined to be 20.6 ± 10.1 and 69.6 ± 29.3 μg/L, respectively (Figure 2). Chloroform constituted 96% of the total THM concentration in SP-A and 99% in SP-B. The other THM species, DCBM, DBCM, and bromoform, were determined to be lower than 2.0 μg/L. Based on Spearman correlation analysis, a positive correlation was observed between TOC and THM concentrations in SP-A and SP-B (*p* < 0.01, Table S3). TOC concentration, swimmer load, and chlorine dose were reported to affect THM formation.^39,40,43−45^ Replacement water amount and frequency^37^ and disinfectant dose^46,47^ and type^48^ also affected the formation and speciation of THMs. The total THM concentration was reported to range from 26 to 213 μg/L in 23 indoor SPs^49^ and from 19 to 146 μg/L in 11 indoor SPs,^50^ from 18 to 114 μg/L in 15 indoor SPs in Canada,^51^ from 13 to 46 μg/L with an average value of 15.8 ± 7.2 μg/L in 20 different SPs,^52^ from 105 to 130 μg/L in Ireland,^53^ and from 26 to 65 μg/L in Thailand.^54^ In another study conducted in France, the THM concentration in an indoor swimming pool was determined to be 50 μg/L.^45^ Thacker and Nitnaware^55^ reported that the THM concentration in India reached 355 μg/L. The average THM concentrations were determined to be 40, 79, 132, and 23 μg/L in monitoring studies conducted in Italy, Australia, England, and Korea, respectively.^56−59^ In the present study, THM concentrations of the monitored pools were determined to be similar with the literature. This is probably because the TOC and free chlorine concentrations were higher in SP-B and THM formation is higher than that in SP-A.^39,40,45^

**Figure 3:**
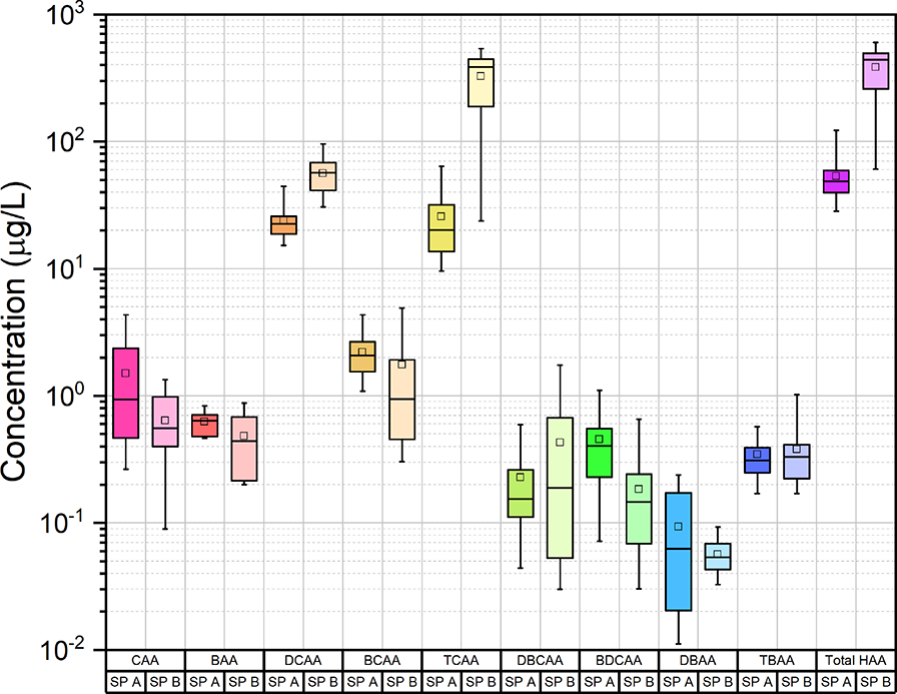
HAA concentrations of swimming pool waters.

HAAs, which were the most studied DBPs after THMs, accumulate in pool waters due to their non-volatile structures and pool treatment equipment being inefficient in their removal.^11,41,60^ HAAs were determined to be at higher concentrations compared to other DBPs in the pool waters investigated in this study. The average HAA concentrations in SP-A and SP-B were measured to be 55 and 385 μg/L, respectively (Figure 3). There was a correlation between TOC and HAA concentrations in SP-A (*p* < 0.05) and SP-B (*p* < 0.01) (Table S3). Chlorinated HAAs were frequently determined than the other HAAs. Among the HAA species, the highest concentrations were determined for TCAA.^14^ The average pool water TCAA, DCAA, CAA, and BCAA concentrations in SP-A were determined to be 25, 24, 1, and 2 μg/L, respectively. In SP-B, the average concentrations of these HAA species were 324, 56, 1, and 2 μg/L, respectively. The concentrations of other HAAs (CAA, BAA, BCAA, DBCAA, BDCAA, DBAA, TBAA) were below or near the MDL. DCAA and TCAA concentrations in SP-A constituted 45 and 50% of total HAAs, respectively, and 15 and 85% of total HAAs in pool B, respectively. Anthropogenic pollutants, albumin, aspartic acid, histidine, and citric acid increased the formation of DCAA and TCAA.^37,61^ During the monitoring campaign, variations in the TOC concentrations were determined to be low with coefficient of variation (CV) values of 14.5 and 34.7% in SP-A and SP-B, respectively. Similarly, the CV values for HAA concentrations were determined to be 33.2 and 43.7%, respectively. The relatively lower CV values might be related to the dilution with the regular replacement of SP water with freshwater in SP-A. On the other hand, the TOC and HAA concentrations in SP-B were increased with time probably due to the irregular pool water replacement strategy.

**Figure 4:**
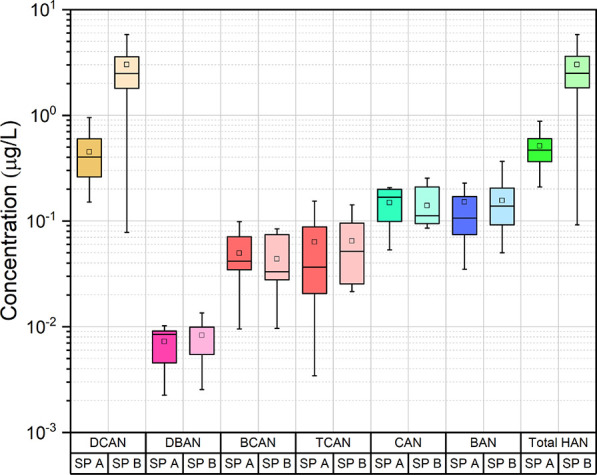
HAN concentrations of swimming pool waters.

The HAN concentrations shown in Figure 4 were determined to be much lower than THM and HAA concentrations, which may be due to rapid degradation and low formation at pH > 7.^62^ The maximum HAN concentration in SP-A was determined to be 2 μg/L, but HNM species cannot be detected in pool water. DCAN was reported to be the dominant HAN species in chlorinated swimming pools.^13,37,63^ In SP-A, DCAN was the dominant HAN species accounting for 81% of the total HAN concentration. The average HAN concentration in SP-B was determined to be 2.9 μg/L, and HNM species cannot be detected similar to SP-A. DCAN was the dominant species accounting for 96% of the total HAN concentration. When the pH of the swimming pool was >7, the HAN concentration tended to decrease due to the fact that the HAN species are degraded and transformed to HAAs.^11^ Therefore, HANs were the less frequently detected DBPs due to pH being >7 in both SPs. The reason that HAN species can only be detected in SP-B might be its higher DON concentrations. Formation of HANs depends on their precursor levels in water. The DON contents were determined to be 0.54 and 1.35 mg/L in the water of SP-A and SP-B, respectively. This finding can explain the detection levels of HANs in SP-B.

**Figure 5:**
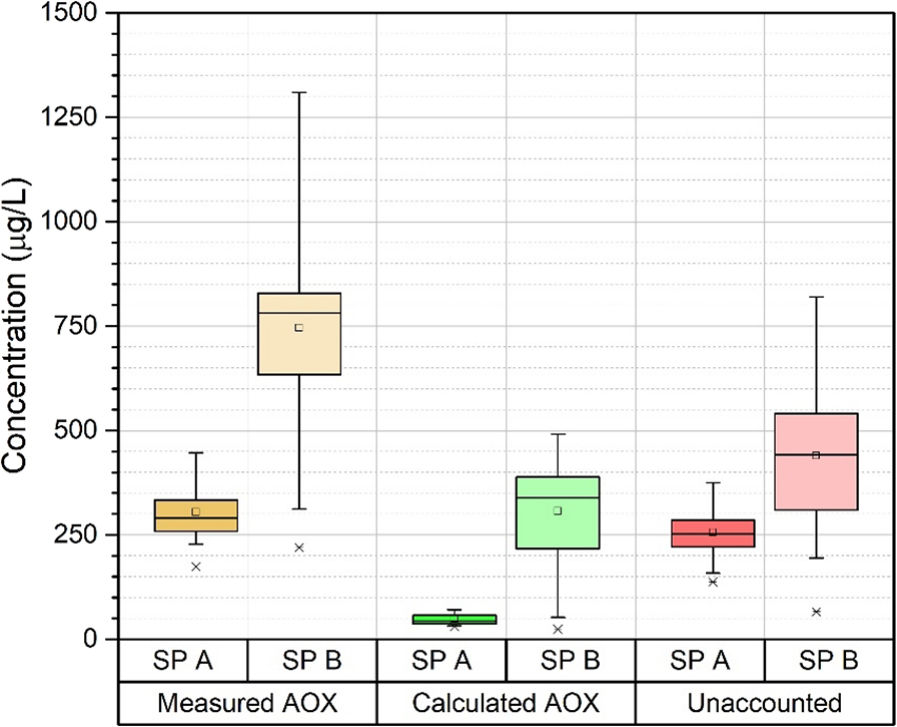
AOX concentrations of swimming pool waters.

AOX represents the amount of halogenated (chlorinated, brominated, and iodinated) organic compounds in the water.^64^ Therefore, the measured AOX includes the DBPs (THM, HAA, and HAN) analyzed within the scope of the study. In this context, the amount of AOX from the measured DBPs was calculated and compared with the determined AOX levels. The difference (measured AOX-calculated AOX) shows the unaccounted amount of halogenated organic matter. Briefly, AOX provides an overview of all by-products that may be present. Peng et al.^26^ and Kimura et al.^65^ suggested that the organic halogen level is a suitable indicator of halogenated DBPs in disinfected waters. The measured and calculated AOXs of SP-A and SP-B are presented in Figure 5. The average AOX concentration in SP-A was determined to be 304 μg/L as Cl^–^. The chlorinated DBPs measured in SP-A accounted for 48 μg/L as Cl^–^. Therefore, an amount of 256 μg/L as Cl^–^ on average is the unaccounted (undetectable/unknown) halogenated compounds. The amount of unaccounted AOX did not vary widely in SP-A during the 48-week campaign (Figure S5). There is a slight increase in the amount of AOX measured over time. This increase in AOX is thought to result from the cumulative increase in pool attendance (*p* < 0.01). The average AOX measured in SP-B was 746 μg/L as Cl^–^, and the unaccounted AOX concentration tended to increase gradually during the monitoring campaign (Figure S5). The number and profile of swimmers, the amount of free chlorine, and UV disinfection were reported to cause the three to four times increase in the AOX levels.^66−69^ They also showed that advanced oxidation processes changed the structure of organic matter by causing AOX to increase. Thus, an increase in chlorinated by-products was observed.^70^ The average amount of AOX from the measured DBPs was calculated to be 307 μg/L as Cl^–^. The average difference between the measured and calculated is 439 μg/L as Cl^–^ due to the undetectable/unknown chlorinated by-products in the pool water. The increase in the difference during monitoring probably resulted from the accumulation of non-volatile unknown/unmeasurable chlorinated by-products in the water.

### 3.3 Correlation and Regression Analyses

Strong linear correlations between total THM and HAA, total THM and UV_254_, and HAA and UV_254_ were observed in chlorinated surface waters in Turkey.^71^ Also, THM and HAA concentrations were reported to be moderately correlated with AOX concentrations. However, relationships between these variables have not been investigated in swimming pools. The correlation matrix (Table S4) lists values of correlation coefficients (*R*) between analyzed parameters. Inverse correlations were determined between DBPs and SUVA values (−0.74 for THM, −0.62 for HAN, and −0.72 for HAA). THM, HAN, and HAA were significantly correlated with TOC, DON, and AOX (*R* = 0.65–0.92). Correlations between DBPs and pH, free Cl, UV_254_, and number of swimmers were not significant. Continuous pH and free Cl control mechanism to ensure compliance with limit values might be the reasons for the non-correlation. HNMs were not included in the analysis due to a high percentage of values below the detection limit (BDL). Strong significant linear relationships (*r*^2^ = 0.63–0.77, *p* < 0.01) were determined between THM, HAA, and HAN concentrations (Figure S6) in accordance with the literature.^72,73^ Source characteristics are known to include the determining factors in formation of DBPs.^18,41,44,74,75^ THM concentrations were strongly related to SUVA (*r*^2^ = 0.54, *p* < 0.01), DON (*r*^2^ = 0.60, *p* < 0.01), TOC (*r*^2^ = 0.67, *p* < 0.01), and AOX (*r*^2^ = 0.85, *p* < 0.01) levels while free chlorine (*r*^2^ = 0.11, *p* < 0.01) and number of swimmers (*r*^2^ = 0.04, *p* = 0.05) were not (Figure S7). Similar to total THM, total HAA was found to be correlated with SUVA, DON, TOC, and AOX with *r*^2^ values of 0.52, 0.59, 0.42, and 0.76 (*p* < 0.01), respectively, but not to pH (*r*^2^ = 0.08, *p* = 0.01), free chlorine (*r*^2^ = 0.17, *p* = 0.23), and number of swimmers (*r*^2^ < 0.01) (Figure S8). While the correlations of SUVA with THM and HAA concentrations were relatively strong, it was not the case with HAN (*r*^2^ = 0.38, *p* < 0.01). Nevertheless, the total HAN concentrations were also related to DON, TOC, and AOX with *r*^2^ values of 0.53, 0.63, and 0.72 (*p* < 0.01), respectively (Figure S9) but not to free chlorine and number of swimmers (*r*^2^ = 0.02, *p* > 0.05). Adequacy of all SLR models was checked for residual normality, randomness, and constant variance, while the SLR models between free Cl vs THM, HAA, and HAN and swimmers vs THM, HAA, and HAN were not adequate due to the low linearity between parameters, non-normal, biased, and heteroscedastic residuals. Despite the heteroscedastic residuals of other predictive SLR models of DBPs, residuals are distributed normal; the models are significant at the 0.05 level. The proposed models might be used for estimation THM, HAA, and HAN concentrations with simple parameters such as DON and especially AOX because it was the best predictive variable for THM, HAA, and HAN.

## 4 Conclusions

Swimming pools that are regularly disinfected with chlorine and other disinfectants can have higher levels of DBPs, which can pose a potential health risk to swimmers and pool staff. Therefore, it is important to regularly monitor and maintain toxic compounds in swimming pools to ensure the safety of all swimmers and staff. By taking proactive measures to reduce DBPs, swimmers can enjoy their time in the pool without having to worry about potential health risks.

Prediction of DBPs in swimming pools is an important factor in maintaining the safety of pools. By using predictive models and monitoring tools, pool operators can accurately estimate the levels of DBPs in the pool and take proactive measures to reduce them. By taking these steps, pool operators can ensure that swimmers are able to enjoy their time in the pool without having to worry about potential health risks.

UV_254_ absorbance is generally identified as an indicator of the precursors of DBPs; however, it provides limited information about DBP formation. To gain a better understanding, SUVA_254_ levels and TOC concentrations need to be calculated together. The DON concentration is an important factor to consider when assessing water quality; in swimming pools, increases over time, which might be due to the accumulation of nitrogenous anthropogenic contaminants.

The accumulation of non-volatile DBPs in pool water was indicated by the increasing AOX concentrations in swimming pools. This is an important issue because most of those by-products can have a detrimental effect on human health. Therefore, reducing the amount of precursors and non-volatile DBPs is important in reducing AOX levels.

High THM and HAA levels observed in both sample pools indicate that the swimmers and pool staff might be exposed to significant doses. While HAA occurrence was determined to be higher than those other targeted compounds, HAN formation occurring at low levels that were dominated by DCAN. DCAA and TCAA were the dominant compounds of HAAs. TOC, DON, SUVA_254_, and AOX concentrations can be used to predict THM, HAN, and HAA concentrations using SLR without complex analytical experiments. Unaccounted AOX concentrations and their relation to the total THM, HAA, and HAN concentrations suggest that the non-targeted halogenated compounds might be important in public health mitigation efforts for swimmers and pool staff.

## Supporting Information Available

Extraction and analysis methods of DBPs; results of normality tests and correlation tests; linear fitting plots; temporal monitoring results of EC, DON, TOC, and AOX (PDF)
