# Supplementary material for: Halogenated By-Products in Chlorinated Indoor Swimming Pools: A Long-Term Monitoring and Empirical Modeling Study
Source: ACS Omega. 2023 Mar 15;8(12):11364–72. doi: 10.1021/acsomega.3c00091 (PMC10061505; doi:10.1021/acsomega.3c00091)
Supplement: Supplementary file 1 — ao3c00091_si_001.pdf [file ao3c00091_si_001.pdf]

Supporting Information

Halogenated By-Products in Chlorinated Indoor Swimming Pools: A Long-Term Monitoring and Empirical Modelling Study

Mesut GENISOGLU[a*], Mert MINAZ[bc], Ertac TANACAN[b], Sait Cemil SOFUOGLU[a],

Sehnaz Sule KAPLAN-BEKAROGLU[b], Amer KANAN[d], Nuray ATES[e],

Tugba SARDOHAN-KOSEOGLU[f], Nevzat Özgü YIGIT[b], Bilgehan Ilker HARMAN[b*]

[a]*Department of Environmental Engineering, Izmir Institute of Technology, Izmir, Turkey*

[b]*Department of Environmental Engineering, Suleyman Demirel University, Isparta, Turkey*

[c]*Department of Aquaculture, Recep Tayyip Erdoğan University, Rize, Turkey*

[d]*Department of Environment and Earth Sciences, Al-Quds University, Palestine*

[e]*Department of Environmental Engineering, Erciyes University, Kayseri, Turkey*

[f]*Department of Biomedical Engineering, Applied Sciences University of Isparta, Isparta, Turkey*

***Corresponding Authors:** *mesutgenisoglu@iyte.edu.tr, ilkerharman@sdu.edu.tr*

SI 1. Extraction and Analysis Methods of DBPs

SI1 a. THM and HAN analysis

THM and HAN analysis were performed according to USEPA 551.1 liquid-liquid extraction method and measured by gas chromatograph (USEPA, 1996). Glass bottles with 40 ml PTFE caps were used for extraction. 8 ml of 99.8% HPLC purity MTBE was rapidly added as the organic solvent phase. Subsequently, 8.5 g of $NaSO_4$ was added for both easy separation of THMs by increasing ionic intensity in the water phase and reducing the solubility of MTBE in water. Extraction bottles were closed and mixed horizontally for 15 minutes at 300 rpm. After 15 minutes of mixing time, it was left for 15 minutes to separate the phases and transferred from the MTBE phase to 2 ml GC vials using glass pipettes. 1000 µg/L buffer stock solution was prepared from the main stock of THM and HAN and 6 calibration standards were prepared at 1, 5, 10, 25, 50 and 75 µg/L concentrations according to various dilution rates. Samples and standards were prepared according to the same method (USEPA 551.1) and analyzed using the Agilent 6890 GC with ECD and Agilent Chem-Station software. The method details used in the GC are described below.

Column properties: Inner Diameter: 0.25 mm, film thickness: 0.25 µm, length: 30 m, DB-5 (J&W Scientific).

Temperature program: initial furnace temperature: 35 °C, 125 °C in increments of 10 °C/minute, and 10 minutes at 125 °C, 300 °C in increments of 30 °C/minute. Inlet temperature was 200 °C and detector temperature was 290 °C.

Injection volume: 3 µL.

Gases: ultra-high purity helium was used as carrier gas and high purity nitrogen gas was used for make-up gas.

Flow rate of gases: carrier gas + make-up gas: 60 ml/min, carrier gas: 1.4 ml/min.

SI 1 b. HNM Analysis

USEPA 551.1 (USEPA, 1995) method was used in HNM analysis by making some modifications for liquid-liquid extraction. After adding 10 ml of methyl tertiary butyl ether (MTBE) as organic solvent phase for 10 ml of sample, 3 g of sodium sulfate (Na_2SO_4) was added to the sample to increase ionic strength and 1 g of copper sulfate ($CuSO_4$) was used to facilitate phase separation. After 30 minutes of mixing time, the samples are left for 15 minutes to separate the phases, and then 2 ml gas chromatography from the MTBE phase using glass pipettes.

After 30 minutes of mixing time, the samples were left for 15 minutes to separate the phases, and then transferred to 2 ml gas chromatography (GC) sample bottles from the MTBE phase using glass pipettes. Calibration standards were prepared using HNM main stocks and extraction was completed using the same method as the samples. The prepared samples were analyzed using the Agilent 6890 GC with the electron capture detector (ECD) and the software "Agilent Chem-Station".

Column properties: Inner Diameter: 0.25 mm, film thickness: 0.25 µm, length: 30 m, DB-5 (J&W Scientific).

Temperature program: initial furnace temperature: 35 °C, 140 °C in increments of 10 °C/minute, 300 °C in increments of 30 °C/minute. Inlet temperature was 117 °C and detector temperature was 297 °C.

Injection volume: 2 µL.

Gases: ultra-high purity helium was used as carrier gas and high purity nitrogen gas was used for make-up gas.

Flow rate of gases: carrier gas + make-up gas: 62.3 ml/min, carrier gas: 1.6 ml/min.

SI 1c. HAA analysis

HAA analysis was performed according to USEPA 552.2 liquid-liquid extraction method. Glass bottles with 40 ml PTFE caps were used for extraction. 25 ml of chlorinated samples were taken. 2 ml of sulfuric acid and organic solvent were rapidly added to the sample as 4 ml of 99.8% HPLC purity MTBE. 11 grams of Na_2SO_4 was added both for easy separation of HAAs by increasing ionic intensity in the water phase and to decrease the solubility of MTBE in water. Extraction bottles were closed and mixed horizontally at 200 rpm for 3 minutes. After 3 minutes of mixing time, it was left for 5 minutes to separate the phases and 3 ml was taken from MTBE phase using automatic pipettes and placed in 15 ml glass bottles. Subsequently, 3 ml of 10% acidic methanol solution was added to this MTBE phase. For 100 ml of 10% acidic methanol solution, 10 ml of sulfuric acid was added to 90 ml of methanol. 15 ml glass bottles were kept in a 50 °C water bath for 2 hours. After 2 hours, the glass bottles were taken from the water bath and left to cool at room temperature. Salt crystals were formed at the bottom of some of the bottles due to the samples taken from the 50 °C water bath to cool at room temperature. 7 ml Na_2SO_4 (150 g/L) was added to the bottles for easy separation of the MTBE phase. After waiting a bit for precipitation and phase separation, 7 ml phase formed at the

bottom was taken. 1 ml of saturated sodium bicarbonate ($NaHCO_3$) solution was added to the 3 ml MTBE phase remaining in the bottle and mixed 5 times with each mixing for 5 seconds. The bottle cap was loosened to release the gas formed after the first mixing. Two phases formed after mixing. 1 ml was taken from the MTBE phase formed above and transferred to GC vials. 8 mixed calibration standards (0.1 - 2000 µg / L) varying in wide concentration ranges were prepared for each HAA type according to various dilution rates using mixed HAA main stock. Samples and standards were prepared according to the same method (USEPA 552.2) and analyzed using the Agilent 6890 GC with ECD and Agilent Chem-Station software.

Column properties: Inner Diameter: 0.25 mm, film thickness: 0.25 µm, length: 30 m, DB-5 (J&W Scientific).

Temperature program: initial furnace temperature: 45 °C and 20 minutes in 45 °C, 140 °C in increments of 5 °C/minute, 165 °C in increments of 15 °C/minute and 3 minutes in 165 °C. Inlet temperature was 200 °C and detector temperature was 290 °C.

Injection volume: 2 µL.

Gases: ultra-high purity helium was used as carrier gas and high purity nitrogen gas was used for make-up gas.

Flow rate of gases: carrier gas + make-up gas: 62.2 ml/min, carrier gas: 2.2 ml/min.

Table S1. Normally Test of TOC, Attended Swimmers, AOX, Total THM and Total HAA for SP-A and SP-B

**Tests of Normality in SP-A**			
	Kolmogorov-Smirnov[a]		
	Statistic	df	Sig.
TOC	,140	48	,019
Attended Swimmers	,251	48	,000
AOX	,151	48	,008
Total THM	,168	48	,002
Total HAA	,160	48	,004
**Tests of Normality in SP-B**			
TOC	,148	48	,010
Attended Swimmers	,269	48	,000
AOX	,163	48	,003
Total THM	,070	48	,200*
Total HAA	,154	48	,006

*. This is a lower bound of the true significance.

a. Lilliefors Significance Correction

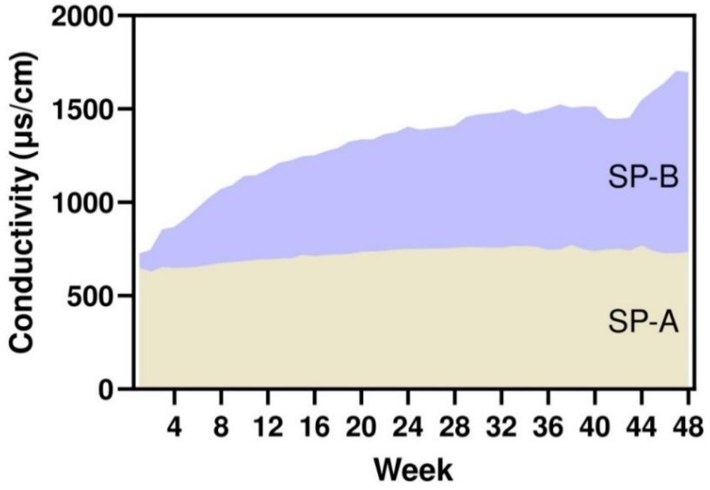

Figure S1. Electrical Conductivity by Time in SP-A and SP-B

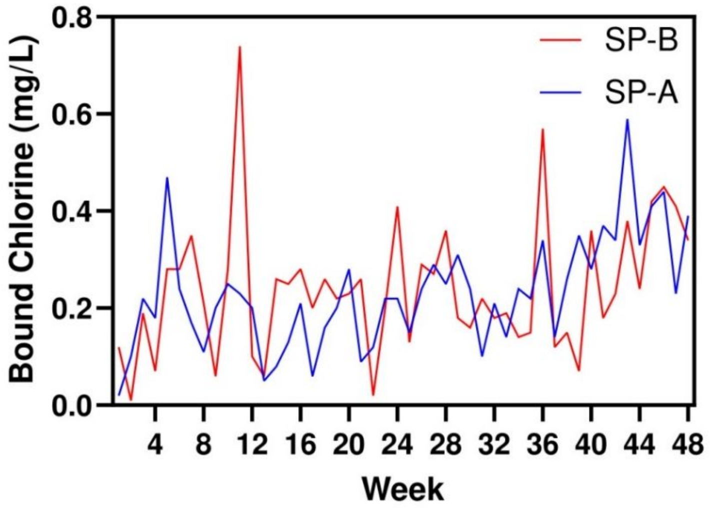

Figure S2. Bound Chlorine by Time in SP-A and SP-B

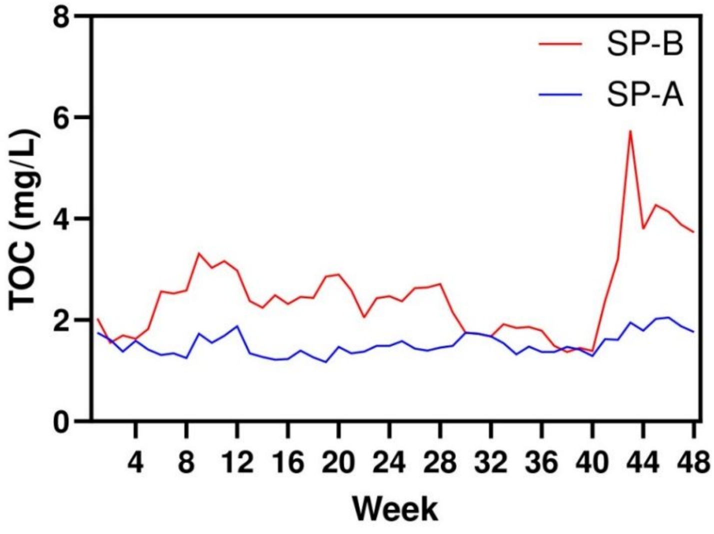

Figure S3. Total Organic Carbon by Time in SP-A and SP-B

Table S2. Correlation Analysis between TOC and Attended Swimmers in SP-A and SP-B

Correlations				
**SP-A**			**Attended Swimmers**	**TOC**
Spearman's rho	Attended Swimmers	Correlation Coefficient	1,000	
		Sig. (2-tailed)	.	
	TOC	Correlation Coefficient	,463**	1,000
		Sig. (2-tailed)	,001	.
**SP-B**			**Attended Swimmers**	**TOC**
Spearman's rho	Attended Swimmers	Correlation Coefficient	1,000	
		Sig. (2-tailed)	.	
	TOC	Correlation Coefficient	,685**	1,000
		Sig. (2-tailed)	,000	.

**. Correlation is significant at the 0.01 level (2-tailed).

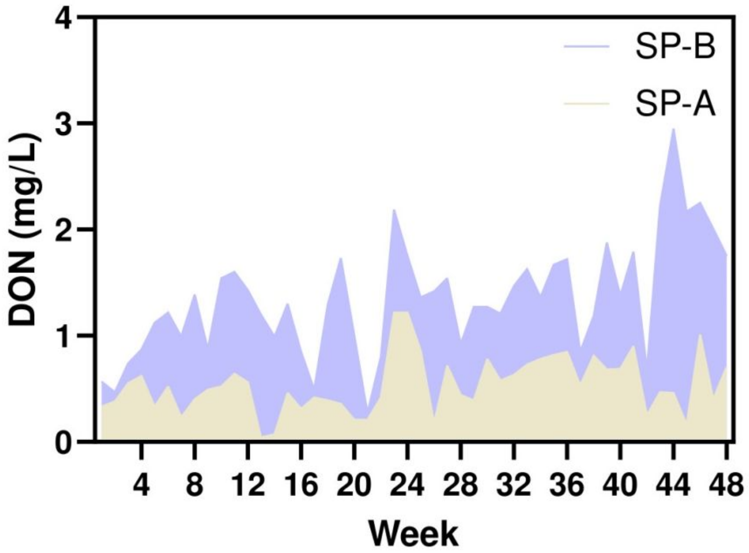

Figure S4. Dissolved Organic Nitrogen by Time in SP-A and SP-B

Table S3. Correlation Analysis between TOC, Total THM and Total HAA in SP-A and SP-B

**Correlations**					
**SP-A**			**TOC**	**Total THM**	**Total HAA**
Spearman's rho	TOC	Correlation Coefficient	1,000		
		Sig. (2-tailed)	.		
	Total THM	Correlation Coefficient	,473**	1,000	
		Sig. (2-tailed)	,001	.	
	Total HAA	Correlation Coefficient	,318*	,160	1,000
		Sig. (2-tailed)	,028	,278	.
**SP-B**			**TOC**	**Total THM**	**Total HAA**
Spearman's rho	TOC	Correlation Coefficient	1,000		
		Sig. (2-tailed)	.		
	Total THM	Correlation Coefficient	,597**	1,000	
		Sig. (2-tailed)	,000	.	
	Total HAA	Correlation Coefficient	,213	,696**	1,000
		Sig. (2-tailed)	,146	,000	.

**. Correlation is significant at the 0.01 level (2-tailed).

*. Correlation is significant at the 0.05 level (2-tailed).

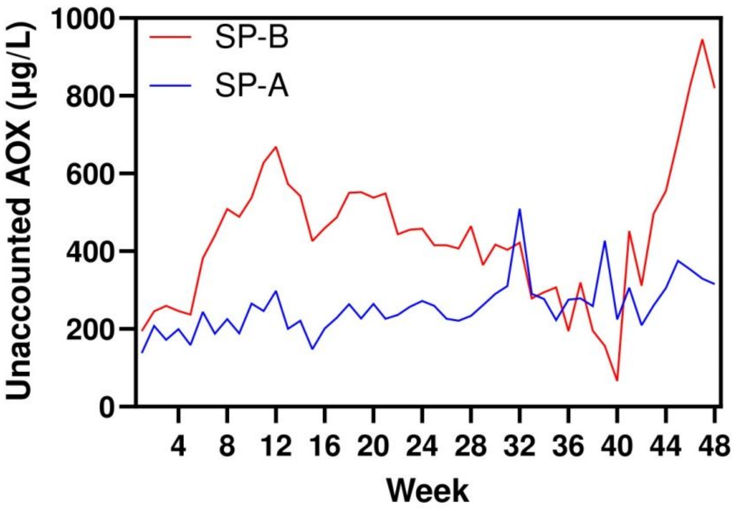

Figure S5. Unaccounted AOX by Time in SP-A and SP-B

Table S4. Correlation table (R) of the analyzed parameters.

	THM	*HAN*	*HAA*	*Free Cl*	*pH*	*UV254*	*SUVA*	*DON*	*TOC*	*Swimmer*	*AOX*
THM	1										
HAN	0.83	1									
HAA	0.88	0.79	1								
Free Cl	0.35	0.14	0.41	1							
pH	-0.36	-0.27	-0.43	-0.07	1						
UV_{254}	-0.45	-0.30	-0.54	-0.37	0.39	1					
SUVA	-0.74	-0.62	-0.72	-0.41	0.34	0.85	1				
DON	0.78	0.73	0.77	0.29	-0.32	-0.43	-0.65	1			
TOC	0.82	0.80	0.65	0.25	-0.11	-0.23	-0.65	0.68	1		
Swimmer	0.20	0.13	-0.03	-0.21	0.02	0.31	0.07	0.05	0.27	1	
AOX	0.92	0.85	0.87	0.37	-0.32	-0.50	-0.76	0.77	0.83	0.07	1

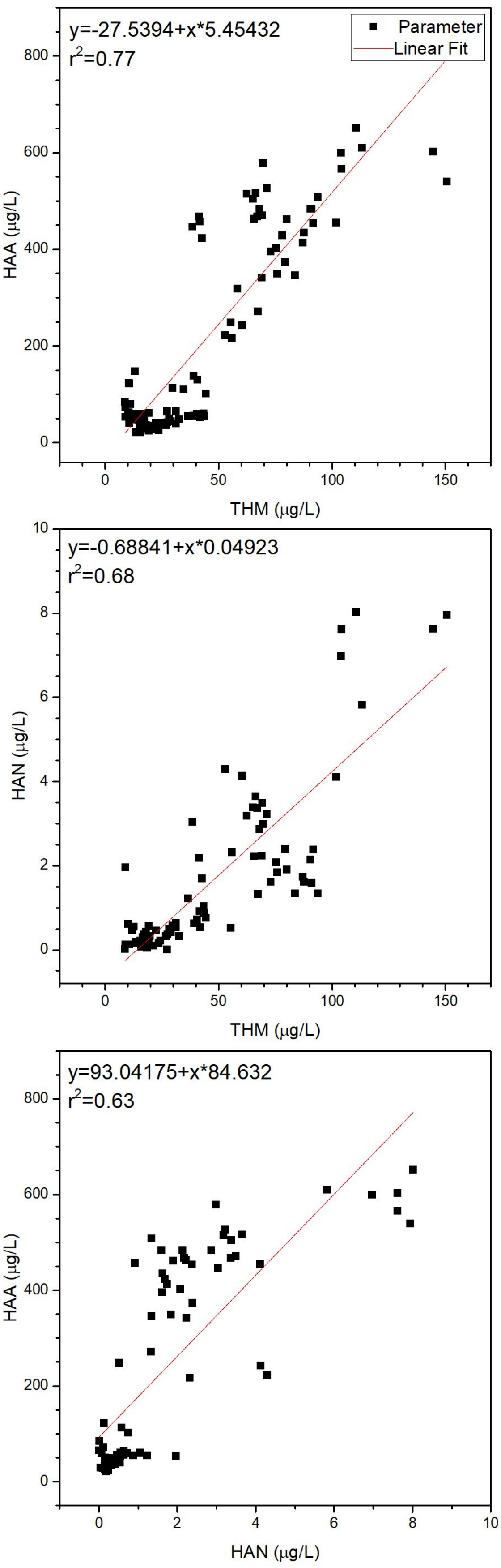

Figure S6. Linear Fitting Plots of DBPs

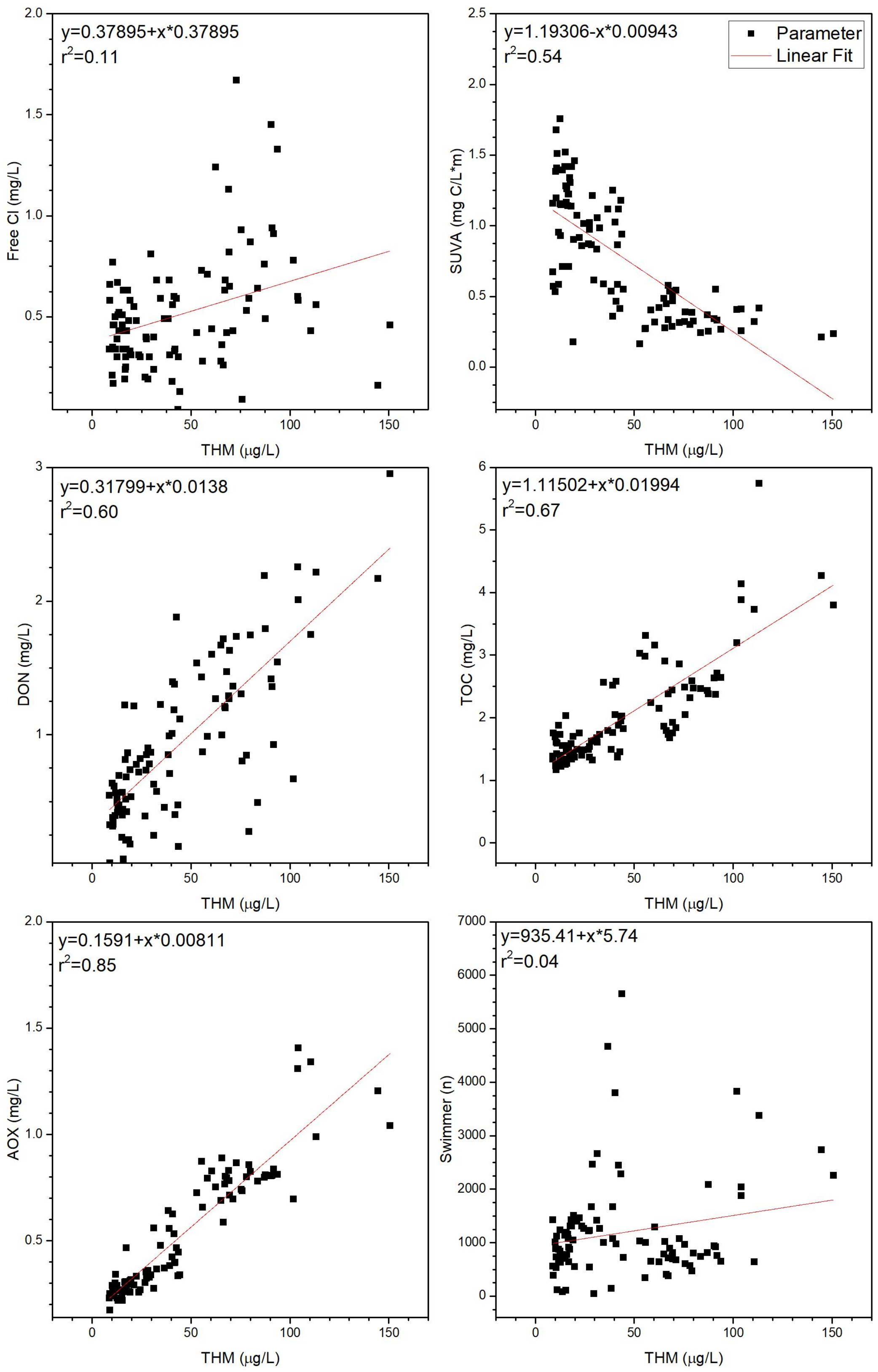

Figure S7. Linear Fitting Plots of THM vs. Parameters

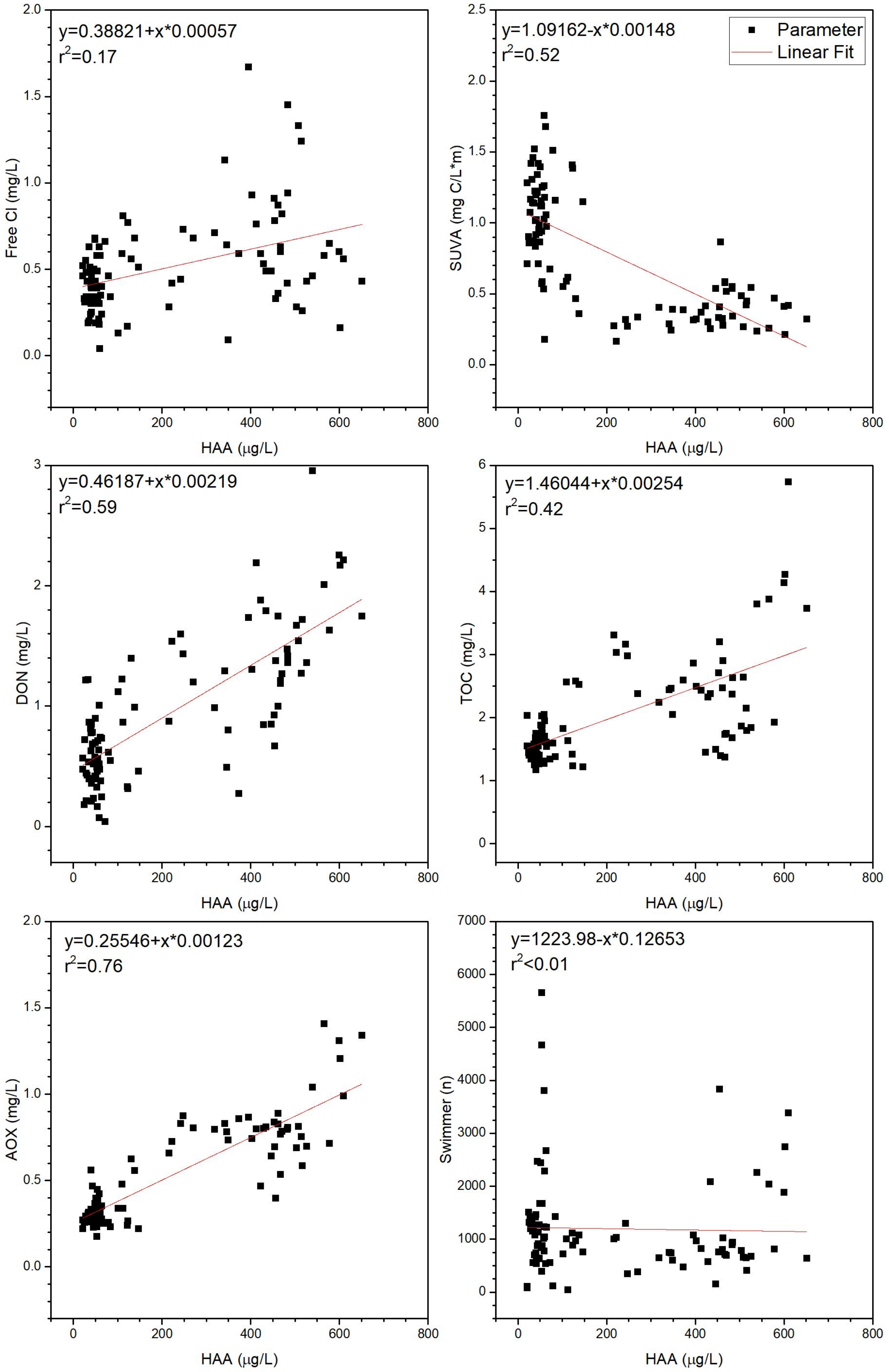

Figure S8. Linear Fitting Plots of HAA vs. Parameters

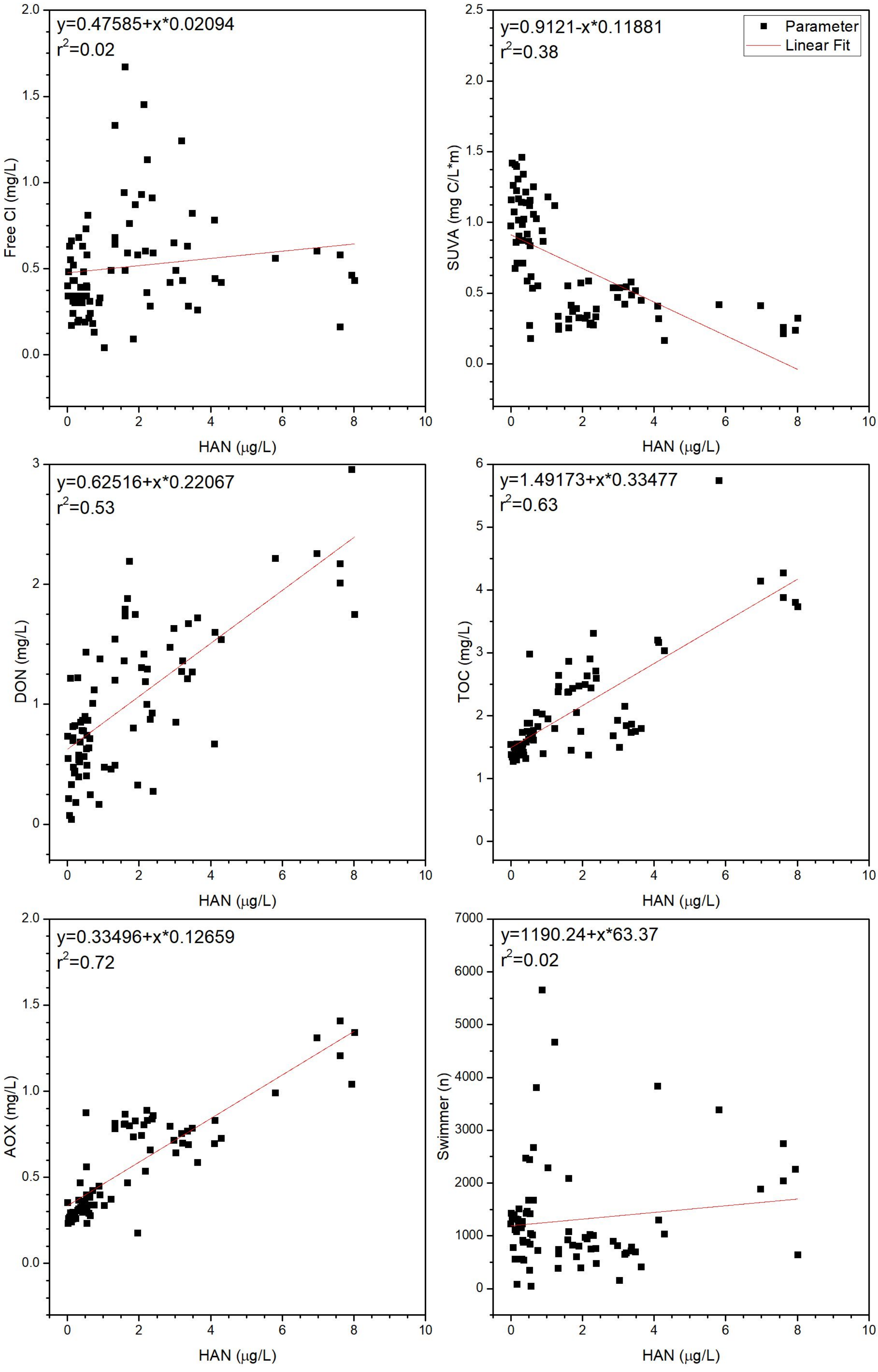

Figure S9. Linear Fitting Plots of HAN vs. Parameters
